# Identification of novel small molecules that inhibit STAT3-dependent transcription and function

**DOI:** 10.1371/journal.pone.0178844

**Published:** 2017-06-21

**Authors:** Iryna Kolosenko, Yasmin Yu, Sander Busker, Matheus Dyczynski, Jianping Liu, Martin Haraldsson, Caroline Palm Apergi, Thomas Helleday, Katja Pokrovskaja Tamm, Brent D. G. Page, Dan Grander

**Affiliations:** 1 Cancer Center Karolinska, Department of Oncology-Pathology, Karolinska Institutet, Stockholm, Sweden; 2 Karolinska High-Throughput Center, Department of Medical Biochemistry and Biophysics, Division of Functional Genomics, Karolinska Institutet Stockholm, Sweden; 3 Chemical Biology Consortium Sweden, Department of Medical Biochemistry and Biophysics, Division of Translational Medicine and Chemical Biology, Karolinska Institutet, Stockholm, Sweden; 4 Department of Medical Biochemistry and Biophysics, Division of Translational Medicine and Chemical Biology, Science for Life Laboratory, Karolinska Institutet, Stockholm, Sweden; University of South Alabama Mitchell Cancer Institute, UNITED STATES

## Abstract

Activation of Signal Transducer and Activator of Transcription 3 (STAT3) has been linked to several processes that are critical for oncogenic transformation, cancer progression, cancer cell proliferation, survival, drug resistance and metastasis. Inhibition of STAT3 signaling has shown a striking ability to inhibit cancer cell growth and therefore, STAT3 has become a promising target for anti-cancer drug development. The aim of this study was to identify novel inhibitors of STAT-dependent gene transcription. A cellular reporter-based system for monitoring STAT3 transcriptional activity was developed which was suitable for high-throughput screening (Z’ = 0,8). This system was used to screen a library of 28,000 compounds (the ENAMINE Drug-Like Diversity Set). Following counter-screenings and toxicity studies, we identified four hit compounds that were subjected to detailed biological characterization. Of the four hits, KI16 stood out as the most promising compound, inhibiting STAT3 phosphorylation and transcriptional activity in response to IL6 stimulation. *In silico* docking studies showed that KI16 had favorable interactions with the STAT3 SH2 domain, however, no inhibitory activity could be observed in the STAT3 fluorescence polarization assay. KI16 inhibited cell viability preferentially in STAT3-dependent cell lines. Taken together, using a targeted, cell-based approach, novel inhibitors of STAT-driven transcriptional activity were discovered which are interesting leads to pursue further for the development of anti-cancer therapeutic agents.

## Introduction

Tumorigenesis is a multistep process in which genetic and epigenetic changes confer growth advantage to the cells driving the progressive transformation of normal cells into malignancy. Unlike healthy cells, cancer cells can grow largely independent of environmental growth signals: they become self-sufficient in growth factor signaling due to the abnormal activation of growth factor receptors, receptor tyrosine kinases (RTK) or other factors [1]. This feature has prompted the development tyrosine kinase inhibitors, which target dysfunctional growth signaling in cancer cells. As targeted anti-cancer therapeutics, RTK inhibitors have revolutionized the cancer drug discovery process and have become valuable weapons in the fight against cancer [2]. RTKs, for example, EGFR, IGFR, VEGF [3–5], non-receptor TKs (such as v-SRC and BCR-ABL) [6, 7] and cytokines activate the transcription factor (TF) STAT3, which in turn drives transcription of genes involved in proliferation, protection from cell death and other processes that are critically important in oncogenesis. As a result, some clinically used inhibitors of TKs can inhibit STAT3 transcriptional activity [8–10]. However, additional TK mutations or switching to alternative TKs can restore STAT3 activation in tumor cells in patients, resulting in acquired resistance to TK inhibitors [11]. Therefore, inhibiting STAT3 activity by targeting STAT3 directly could be a highly beneficial strategy for the successful treatment of cancer.

To date, a number of compounds that inhibit STAT3 phosphorylation and activity have been developed and pre-clinically tested. STATTIC was one of the first small molecules discovered that inhibited function of the STAT3 [12]. However, it has been shown to have multiple off-target effects observed in a variety of studies including our own [13]. It has also been suggested that STATTIC undergoes intracellular modifications, which, together with its small size, makes it capable of binding to a wide range of proteins [14, 15]. The first orally available STAT3 inhibitor, BP-1-102, derived from an earlier STAT3 inhibitor called S3I-201, were developed based on *in silico* docking of the compounds to the SH2 domain of STAT3 [16]. Further investigations of the mechanisms of action of BP-1-102, unfortunately, revealed lack of specificity [17]. Recently, three novel structures were identified in structure-based virtual screenings that aimed at targeting the SH2 domain of STAT3 [18, 19]. The compounds (designated 4a, 4b and B9 respectively) were shown to impact the proliferation rate, viability and the motility of cancer cells with constitutively phosphorylated STAT3. While benzyloxyphenyl-methylaminophenol derivatives 4a and 4b were relatively selective towards IL6/STAT3 pathway, B9 was also able to inhibit the phosphorylation of other STAT family members, illustrating that similarity between the SH2 domains hinders achieving high degree of compound selectivity. Two small molecule inhibitors of STAT3 (OPB-51602 and OPB-31121) have been tested in the early clinical trials [20, 21], however, none has yet advanced to established clinical use [22]. Thus, further efforts towards the discovery and detailed characterization of novel STAT3 inhibitors are required.

Redundant or compensatory signaling roles are also found within the STAT protein family. STAT3 and STAT1 are often activated by the same cytokines and the specificity of the response is determined by the cell context and is modulated in part by reciprocal regulation of STAT3 and STAT1. Although STAT1 has been regarded as a tumor suppressor protein with some functions that oppose the tumorigenic role of STAT3 [23], it can substitute for STAT3 in certain cancer cell lines and adopt a pro-tumorigenic role [24]. Furthermore, an abnormal STAT1 activation and a specific IFN-related gene signature has been linked to chemo-resistance and poor prognosis in cancer patients [25–29]. Altogether, this data describes a complex relationship between STAT1 and STAT3 signaling in cancer that should be considered when developing STAT-targeting compounds.

In this study, a screening system for STAT3 inhibitory compounds was developed, based on monitoring STAT3 transcriptional activity using an interleukin 6 (IL6)-dependent cellular luciferase reporter assay. Conditions were designed to prioritize compounds that directly inhibited STAT proteins rather than upstream kinases or receptors. Hit compounds were confirmed in dose response experiments and further analyzed for their ability to inhibit STAT3 phosphorylation, downregulate STAT3- and STAT1-driven gene expression, and for their potential to impair the viability in STAT3-dependent and -independent cancer cell lines.

## Results

### The development of a cell—based system for the high-throughput screening

For the high-throughput screening assay development, we have selected a reporter assay and two sublines of the colon adenocarcinoma DLD1 cell line [30]. These cell lines consisted of a STAT3-negative subline (referred to as A4) and this same cell line reconstituted with wild-type STAT3 referred to as A4wt. Neither A4 nor A4wt cells are dependent on STAT3 activity, thus inhibiting STAT3 signaling in A4wt cells should not impair cell viability. This system would allow to discern the effects of the compounds on the transcriptional activity from the effects on cell viability. On the other hand, inhibiting luciferase activity in A4wt versus A4 cells would provide an optimal way to selectively investigate STAT3-specific transcriptional activity in a cell-based, high-throughput screening assay, and also would ensure that hit compounds are cell penetrable.

**Fig 1:**
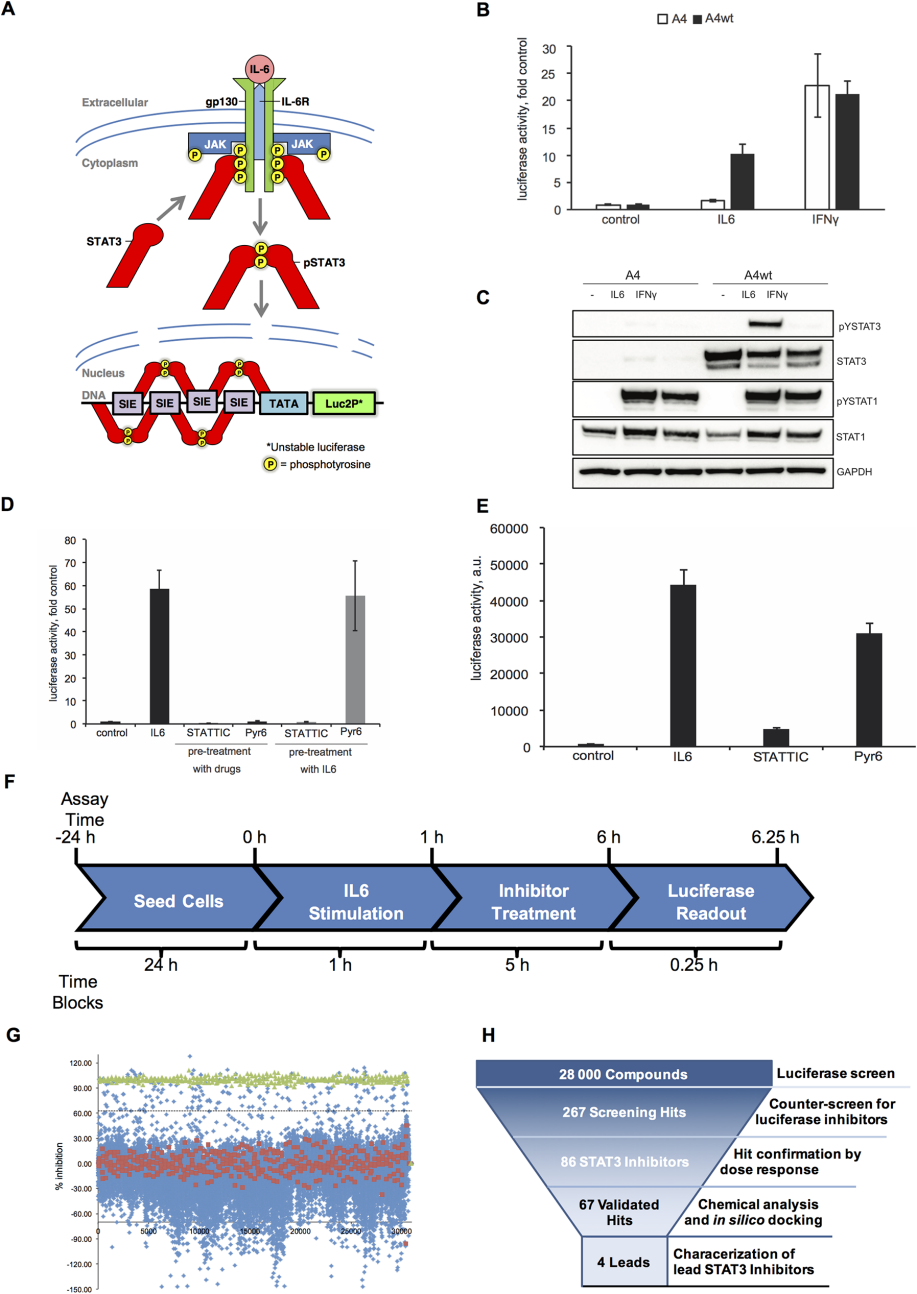
Cellular system for screening of compounds inhibiting STAT3 transcriptional activity. **(A)** Overview of the STAT3 luciferase reporter system. **(B)** A4wt and A4 sublines were transiently transfected with pGL4.27-SIE reporter and pRL-Renilla and either left untreated or were treated with IL6 + IL6R (50ng/mL and 100 ng/mL respectively) or IFNγ (40 IU/mL). After 6h, luciferase activities were measured using Dual-Glo Luciferase assay. The data presented as ratios to the corresponding untreated control. **(C)** A4 and A4wt sublines were treated with IL6 + sIL6R (50ng/mL and 100 ng/mL, respectively) or IFNγ (40 IU/mL) for 30 min. Western blotting analysis was performed with the indicated antibodies. GAPDH was used as a loading control. **(D)** A4wt cells were transfected with pGL4.27-SIE reporter and a stable sub-line A4wt-SIE-6 was selected. Cells were either left untreated (control) or were stimulated with IL6 + sIL6R (50 and 100 ng/mL, respectively) either before treatment with STATTIC (10 μM) or Pyr6 (1 μM), or after, as indicated. Luciferase activity was measured 6h after the first treatment in both cases using SteadyLite^TM^ Plus. The data is presented as fold induction. Error bars represent SE from quadruplicates. **(E)** A4wt cells stably transfected with pGL4.27-SIE reporter were seeded in 384-well plates and pre-treated with IL6 and sIL6R (50 and 100 ng/mL, respectively) for 1h, whereafter treated with STATTIC (10 μM) or Pyr6 (1 μM) for 5h to estimate the suitability of the screening system for the high-throughput screening and calculate the z’-factor. Luciferase activity was measured as in (C). One column of the 384-well plate was used for each treatment. The data is presented as average of raw luciferase activity ± standard deviation. The relevant z’-values are described in the text. **(F)** Outline of the luciferase reporter assay used for high-throughput screening. **(G)** The dot-blot of the results of the primary screening. A4wt-SIE-6 cells stably transfected with pGL4.27-SIE were pre-treated with IL6 and sIL6R and then treated with the drugs. The data are presented as percent inhibition of luciferase activity where the luciferase activity of the cells treated with IL6+sIL6R+mock is regarded as 0% inhibition (red dots), and the activity in cells treated with IL6+sIL6R+STATTIC regarded as 100% inhibition (green dots). The dashed line is the distance of 3 standard deviations from IL6+sIL6R+mock-treated cells. All the library compounds were used at 10 μM. **(H**) The STAT3 inhibitor screening funnel.

In order to define compounds with STAT3 inhibitory activity, we used the stimulation with IL6, a multifunctional cytokine involved in STAT signaling in processes of inflammation and oncogenesis [31]. When IL6 binds to its receptor, the associated Janus Kinases (JAKs) are activated, thus inducing STAT phosphorylation and subsequent transcription factor activity. IL6 can activate both STAT1 and STAT3, although this appears to be tissue and context specific [32]. For the reporter system to monitor STAT3 transcriptional activity, we cloned the STAT-inducible element (SIE, 4X repeated) derived from the promoter of a STAT3-inducible gene, *CFOS* [33], in front of a minimal promoter and a luciferase gene. The latter encodes an unstable luciferase (half-life ≈ 40 min), thus allowing for dynamic monitoring of changes in expression. This process is outlined in **Fig 1A**.

Both STAT3 and STAT1 are able to bind to SIE elements and, thus, drive this reporter [34]. Indeed, as shown in **Fig 1B**, IL6 treatment induced a significant activation of the reporter in the STAT3 reconstituted A4wt subline (about 10-fold compared to the untreated control), whereas only a slight increase of luciferase activity was observed in the STAT3-negative A4 subline. This difference occurred in spite of the fact that IL6 activates STAT1 phosphorylation equally well in both A4 and A4wt cells (**Fig 1C**). In contrast, IFNγ induced luciferase activity in both A4 and A4wt cells (**Fig 1B**). This is consistent with IFNγ-mediated STAT1 phosphorylation as depicted in **Fig 1C**. Thus, IL6 preferentially activate the reporter through STAT3 signaling, whereas IFN*γ*- via STAT1.

To direct our screening system towards STAT3 inhibitors over inhibitors of its upstream activators (i.e. JAKs, gp130), different co-treatment schemes were tested using the known STAT3 inhibitor, STATTIC [12] and a pan-JAK inhibitor, Pyr6 [35]. IL6-induced activation of JAKs is rapid and transient, whereas STAT3 activity can persist for longer time periods due to retention of phosphorylated STAT3 in the nucleus [36–38]. As expected, pre-treatment of A4wt cells stably transfected with the reporter with either STATTIC or Pyr6 prior to the stimulation with IL6 resulted in the abrogation of the reporter activity (**Fig 1D**). However, when Pyr6 was added 1h after IL6 stimulation, it lost most of its inhibitory effect. In contrast, STATTIC retained its effect even when added 1 h after IL6 stimulation (**Fig 1D, grey bars**). Thus, this latter scheme was selected in order to enrich for compounds which may directly target STAT3.

This screening system was optimized for high-throughput screening. Z’-values were established for the different treatment conditions [39]. As shown in **Fig 1E**, the average luciferase activity of the IL6-treated cells is well-separated from that of the untreated control, as well as from STATTIC-treated cells (Z’-for control vs IL6 and for IL6 vs STATTIC ≈ 0.8) indicating that the assay setup was well-suited for high-throughput applications. Pyr6 did not significantly inhibit the luciferase activity under these conditions, suggesting that other potential JAK inhibitors would also be avoided during the screening.

### From library screening to hit selection

28 000 distinct chemical entities from the Drug-Like Diversity Set from ENAMINE library were used for the screening campaign. STATTIC and Pyr6 were used as internal screening controls and the screening was performed according to the scheme depicted in Fig 1F. The scatterplot of raw luciferase activity measurements is shown in **Fig 1G**. Basal luciferase activity was determined using cells that were treated with IL6 followed by DMSO (**Fig 1G**, red dots). STATTIC-treated cells were used as a positive control and the corresponding inhibition of luciferase activity is plotted as green dots in **Fig 1G.** The dashed line is a detection threshold and represents 3X standard deviations of the assays from the negative control. 267 compounds in total, were regarded as primary hits **(Fig 1G)**.

**Fig 2:**
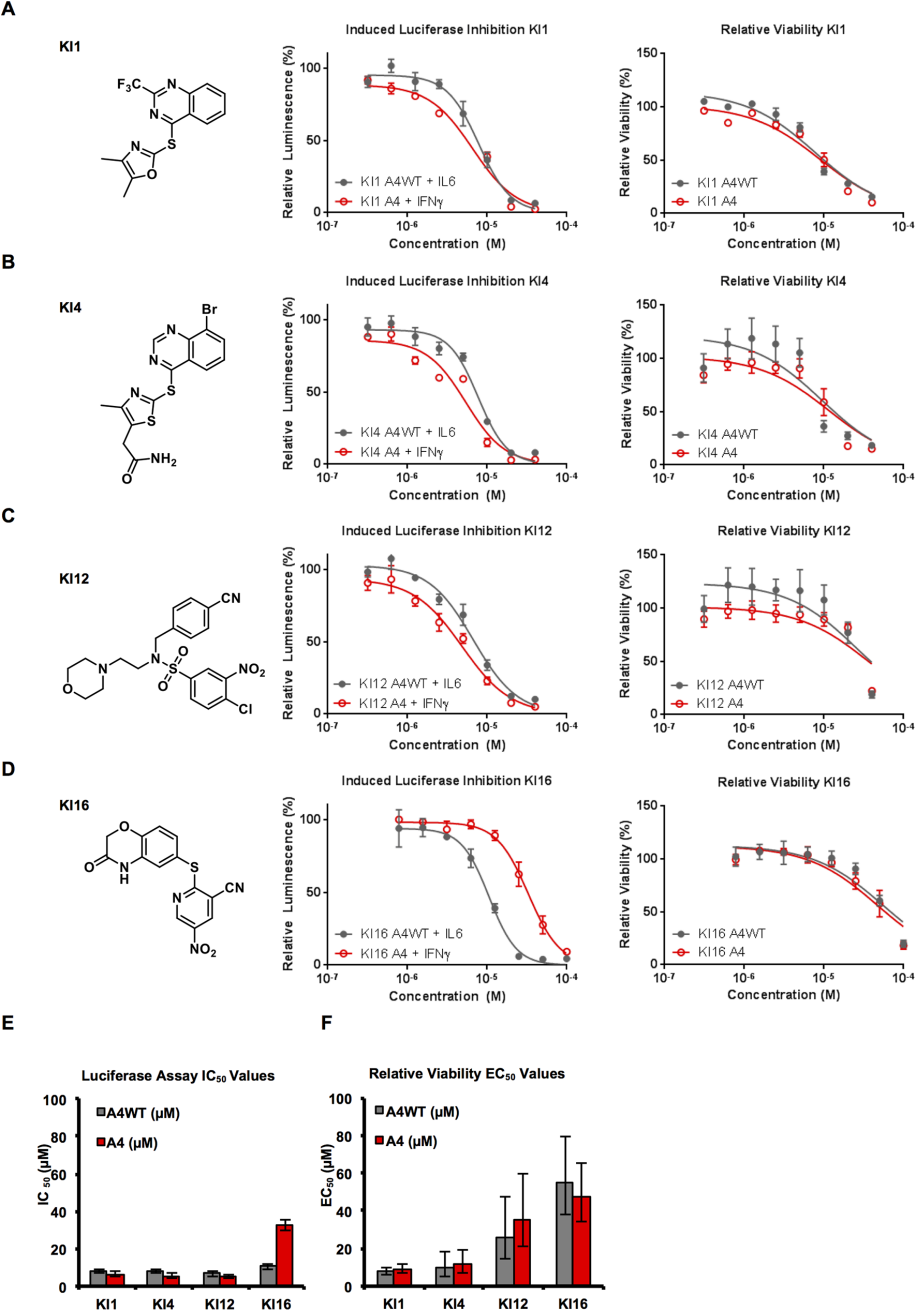
Characterization of the four hit compounds. **(A-D)** Compounds KI1, KI4, KI12 and KI16 and their corresponding activities in the luciferase reporter assays (center) and cell viability assays (right). Luciferase assays were performed in A4wt cells stimulated with IL6 (grey) and in A4 cells stimulated with IFNγ (red). Viability assays were conducted over 48 hours in A4wt cells (grey) and A4 cells (red). **(E)** Luciferase IC_50_ values for lead compounds. Curves were fit using GraphPad and error bars indicate the 95% confidence intervals. **(F)** Cell viability EC_50_ values in A4 and A4wt cells using CellTiterGlo assay (Promega Biotech AB, Sweden). Curves were fit using GraphPad and error bars indicate the 95% confidence intervals.

Primary hits were cherry-picked from the library and tested in a secondary screening, which was performed in duplicates and using three concentrations for each compound at 5, 10 and 20 μM. Compounds that inhibited activity of the luciferase enzyme itself, i.e. “luciferase inhibitors”, were excluded from further investigation. The secondary screen yielded 67 compounds that inhibited the IL6-induced reporter activity by ≥ 25% at 10 μM (**S1 File**). Further profiling of these compounds was performed based on their chemical structures, *in silico* docking and known similarities (this screening funnel is illustrated in **Fig 1H**). Compounds were also excluded if they were suspected to be pan-assay interference compounds [40] or if there was a high likelihood that they were reactive covalent binders. As a result, four compounds were selected for thorough evaluation as STAT3 inhibitors, referred to as KI1, KI4, KI12 and KI16 (**Fig 2A–2D**).

### Characterization of hit STAT3 inhibitors

To further quantify the more detailed effect of the four selected hit compounds on STAT3 signaling, they were evaluated in a luciferase reporter assay at a range of concentrations using A4wt cells stably transfected with the reporter and treated with IL6 (**Fig 2E, grey bars**). The compounds under study were also evaluated for their ability to inhibit STAT1-induced reporter activity in A4 cells treated with IFNγ (**Fig 2E, red bars)**. KI1, KI4, KI12 demonstrated comparable activity against IL6- and IFNγ- induced luciferase activity, while KI16 was more potent in inhibiting IL6-induced luciferase activity in the A4wt cell line. This data suggests that there is little selectivity between STAT1 and STAT3 inhibition for KI1, KI4 and KI12, while KI16 has a potential to be more selective towards STAT3 over STAT1. The compounds were also tested for general toxicity using viability assay. KI1 and KI4 were quite toxic to both A4 and A4wt cell lines at 48h after treatment, while KI12 and KI16 showed toxicity only at higher concentrations (**Fig 2F**). No significant acute toxic effects were detected in these cells within 5 hours of treatment (**S1A Fig)** This data suggested some STAT3-independent effects of the compounds either through inhibition of other STAT family members or through off-target effects.

### Structural analysis of the hit compounds

**Fig 3:**
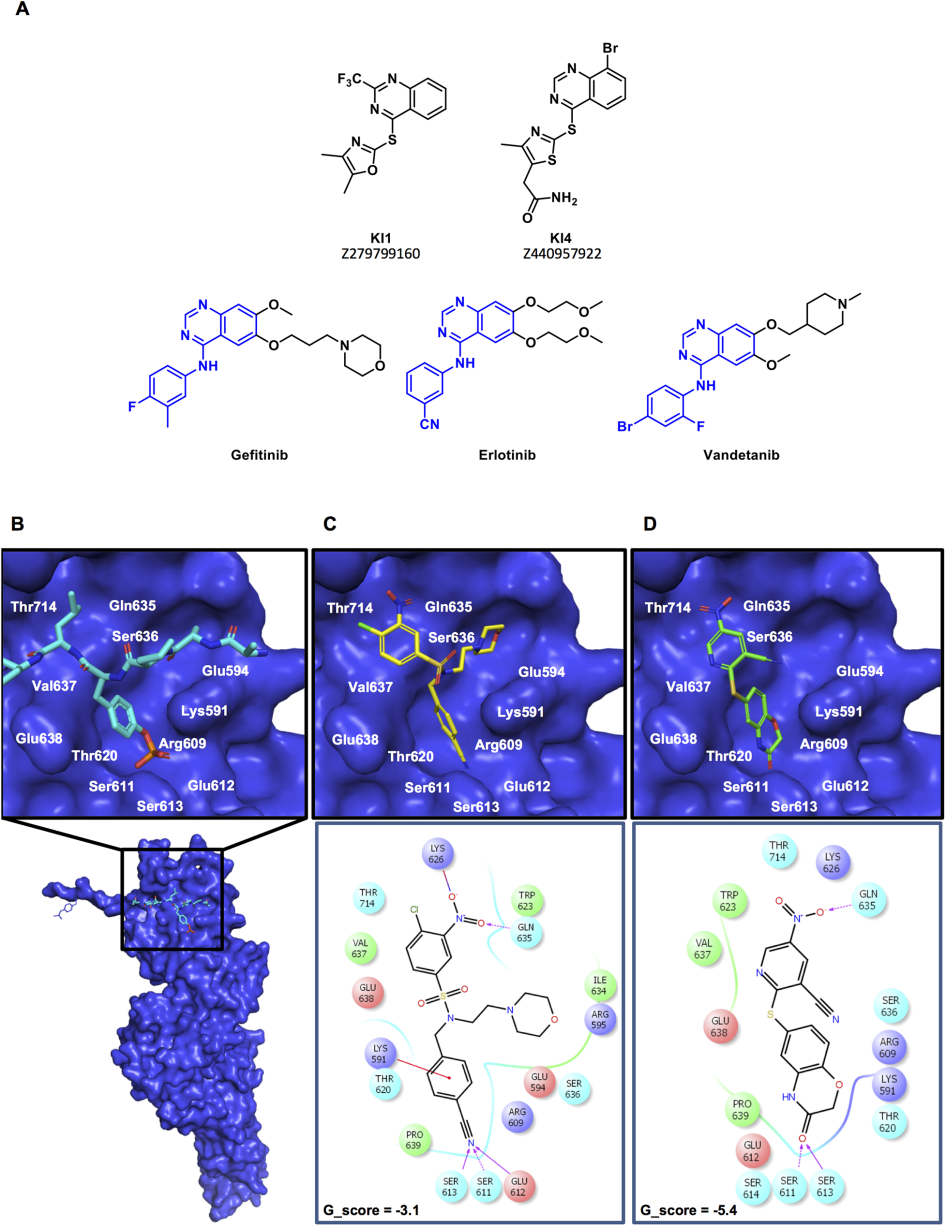
Docking assays and chemical features of the lead compounds. **(A)** Chemical structures of KI1 and KI4. The quinazolone core of KI1 and KI4 is also a common feature of clinically approved EGFR- and VEGFR-targeted therapeutics Gefitinib, Erlotinib and Vandetanib. The pharmacophore portion of the clinically used compounds is highlighted in blue, which is highly similar to the structures of KI1 and KI4. **(B)** STAT3 crystal structure (blue, pdb 1BG1) with residues 702–709 of the opposing monomer shown in cyan. **(C)** (Upper panel) KI12 docked into the STAT3 SH2 domain using GLIDE docking software. (Lower panel) Ligand interaction diagram showing interactions between KI12 and residues on the STAT3 SH2 domain. KI12 hydrogen bonds with Ser611, Ser613 and Glu612 and forms cation-pi type interactions with the positively charged Lys591. An additional hydrogen bond is formed with Gln635 and a salt bridge with Lys626. **(D)** (Upper panel) KI16 docked into the STAT3 SH2 domain using GLIDE docking software. (Lower panel) Ligand interaction diagram showing interactions between KI16 and residues on the STAT3 SH2 domain. KI16 makes hydrogen bonds with Ser611, Ser613 and Gln635.

The structural analysis showed that KI1 and KI4 had similar structural elements to known EGFR and VEGFR inhibitors. The quinazolone core structure (**Fig 3A, blue**), is present in the clinically available drugs Gefitinib, Erlotinib and Vandetanib, and is a known adenine mimetic [41]. The remaining black portion of the molecule extends away from the ATP binding pocket of the receptors and likely contributes to improving solubility in the clinically used derivatives. While the aryl amino substituent of the clinically used compounds is replaced by the 2-thio-thiazole and 2-thio-oxadiazole moieties in KI1 (Z279799160) and KI4 (Z440957922), respectively, it is suspected that this substitution would be well tolerated by the enzyme [42]. Thus, it was suspected that these compounds may be targeting ATP binding pockets of the receptor tyrosine kinases, rather than directly binding to the STAT3 protein. KI12 or KI16 did not have any obvious similarities to known kinase or RTK inhibitors.

Docking analysis was performed using GLIDE [43] docking software (**Fig 3B**). The docking grid on the STAT3 SH2 domain was generated from the binding site of residues 702–709 of the opposing monomer. While both KI12 and KI16 gave docking poses in the STAT3 SH2 domain, KI16 had a much better docking score (KI12: -3.1, KI16: -5.4, **Fig 3C** and **3D**). In fact, compound KI16 was the best scoring in the *in silico* docking studies that we performed on the top 67 hits from the secondary screen. While this compound is not overly complex, the docking results show that KI16 can make hydrogen bonds with Ser611 and Ser613. These serine residues are essential for STAT3-pTyr interactions and it is proposed that by blocking access to these residues KI16 is able to disrupt STAT3 signaling (**Fig 3D**).

In order to further look for evidence of STAT3 SH2 domain binding *in vitro*, compounds KI1, KI4, KI12 and KI16 were analyzed in a fluorescence polarization assay (FPA) [44]. However, none of the hit compounds showed appreciable activity in this assay (data not shown). This may be due to their relatively small size, which makes it difficult to compete with the fluorescent probe which binds the SH2 domain with very high affinity [45]. It is presumed that higher concentrations of inhibitors would be required to observe such activity, however, both KI12 and KI16 showed poor solubility at high concentrations in the FPA buffer. Chemical optimization of KI12 and KI16 could be explored in order to increase the solubility of these compounds and to determine whether similar compounds would bind to the STAT3 SH2 domain.

### The effect of the lead compounds on the IL6-JAK-STAT pathway

**Fig 4:**
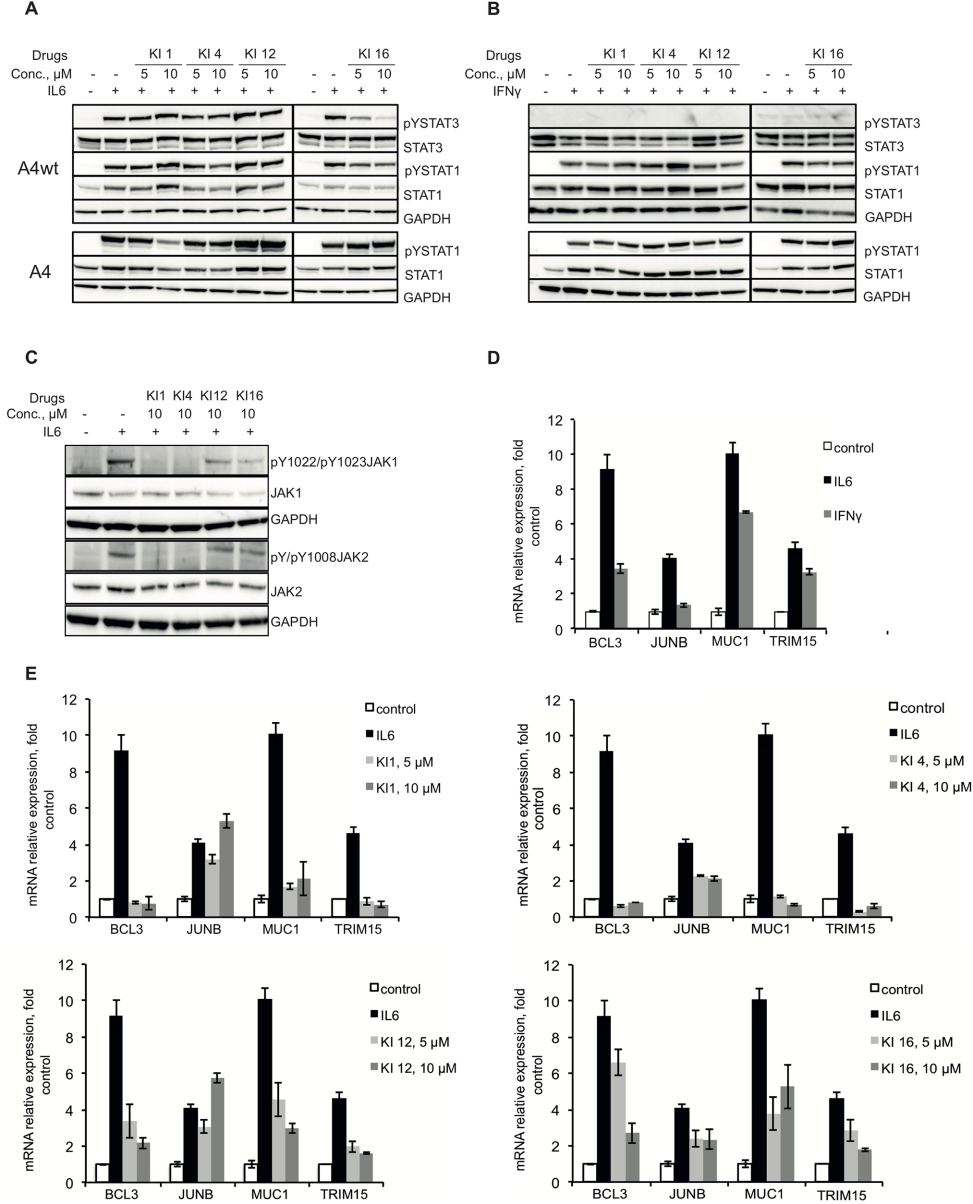
The effect of the lead compounds on the IL6-JAK-STAT pathway. **(A)** A4wt or A4 cell lines were pre-treated with the indicated compounds (5 and 10μM) for 30 min followed by stimulation with IL6+sIL6R (50 and 100 ng/mL, respectively) for 30 min. Levels of phosphorylated STAT3 (pTyr705) and STAT1 (pTyr701) and total levels of these proteins were determined by Western blotting analysis. GAPDH was used as a loading control. Both left and right panels of western blots were performed simultaneously. **(B)** A4wt and A4 cell lines were pre-treated with the indicated compounds (5 and 10μM) for 30 min followed by stimulation with IFNγ (40 IU/mL) for 30 min. Levels of phospho-STAT1 and total STAT1 were determined by Western blotting analysis. GAPDH was used as a loading control. **(C)** A4wt cells were pre-treated with the compounds KI1, KI4, KI12 and KI16 in the concentration of 10 μM and then treated with IL6 + sIL6R (50 ng/mL and 100 ng/mL respectively) for 30 min. The cell pellets were lysed and pJAK1, pJAK2 and total JAK1 and JAK2 levels were assessed by Western blotting. GAPDH was used as a loading control. **(D)** A4wt cells were stimulated with either IL6+sIL6R (50 and 100 ng/mL, respectively) or IFNγ (40 IU/mL) for 4 h. Expression of indicated STAT3 target genes was assessed by qRT-PCR. The data represents mean of duplicates ± SD. **(E)** A4wt cells were pretreated with the indicated compounds (5 and 10 μM) for 30 min followed by IL6+sIL6R stimulation for 4 h (as in **D**). The expression of indicated STAT3 target genes was determined by qRT-PCR. The data is normalized to β-actin expression and presented as fold expression relative to the untreated control. The data represents mean of duplicates ± SD.

In order to understand the mechanism of action of the selected compounds, we investigated their effect on the phosphorylation status of STAT3 (**Fig 4A**), STAT1 (**Fig 4B**) and the upstream kinases JAK1 and JAK2 upon 30 min of IL6 stimulation (**Fig 4C**). KI1 and KI4 inhibited phosphorylation of JAK1 and JAK2 (**Fig 4C**) in line with the structural analysis of KI1 and KI4 that identified similarities with known kinase inhibitors. Interestingly, while JAK phosphorylation was inhibited, neither KI1 nor KI4 significantly inhibited STAT3 phosphorylation (**Fig 4A** and **4B**). KI12 and KI16 had less effect on pJAK1 and no effect on pJAK2 (**Fig 4C**). KI12 did not disrupt STAT3 phosphorylation either, however, potent and dose-dependent inhibition of STAT3 phosphorylation was observed with KI16 (**Fig 4A**). Among the four compounds, only KI1 demonstrated inhibition of STAT1 phosphorylation in response to IL6 stimulation (not with IFNγ) in A4 cells at 10 μM (**Fig 4B**). Inhibition of STAT3 phosphorylation by KI16 together with the docking data, further suggests that this compound may, in fact, be a direct STAT3 binder.

### The lead compounds inhibit IL6—induced STAT- dependent gene transcription

To verify the effect of the hit compounds on the expression of STAT-target genes, we selected a gene panel induced by IL6 in the A4wt cell line (J. Yang, unpublished data), that included *BCL3*, *JUNB*, *MUC1* and *TRIM15*. Promoter analysis using the UCSC genome browser revealed that these genes contain STAT3 binding sites in their promoters. Also, these genes were described to have important functions in cancer cells [46–48]. Therefore, the top inhibitors were analyzed for their ability to disrupt the expression of these STAT3-dependent genes in A4wt cells by qRT-PCR.

As expected, IL6 stimulation induced expression of this panel of genes in the A4wt cell line (**Fig 4D, black bars**); interestingly, IFNγ could also induce *BCL3*, *MUC1 and TRIM15* although to a lesser extent (**Fig 4D, grey bars**), again highlighting the intricate interplay between the STATs where STAT1 is able to regulate the expression of STAT3-driven genes. Each of the four lead compounds inhibited IL-6-induced STAT3-dependent gene expression (**Fig 4E**, grey bars). The compounds did not affect the expression of a house-keeping gene β-actin, which is not under STAT control (**S2B Fig**).

To assess the STAT3 versus STAT1 selectivity of the inhibitors, we first examined *JUNB* expression levels which were upregulated upon IL6 stimulation and not IFNγ and only in the presence of STAT3 (**Fig 4D**, **S2A Fig**). *JUNB* expression induced by IL-6 was only inhibited by KI4 and KI16 among the lead compounds. IFNγ - induced expression of the three STAT3-dependent genes in A4wt cells, most probably driven by STAT1, (**Fig 1B and 1C**), was inhibited KI1 and KI4, whereas KI12 and KI16 were less efficient. IFNγ - induced STAT1-driven genes in A4 cells were inhibited by the compounds, but with KI16 being the least efficient. Notably, *IRF1*, a classical IFNγ-induced gene [49, 50], was not inhibited by KI12 or KI16 (**S2B Fig**) This data is in line with KI1 and KI4 inhibiting upstream kinases, whereas KI12 and particularly KI16 are more selective towards STAT3.

### Inhibiting constitutively activated STAT3 function in cancer cells

**Fig 5:**
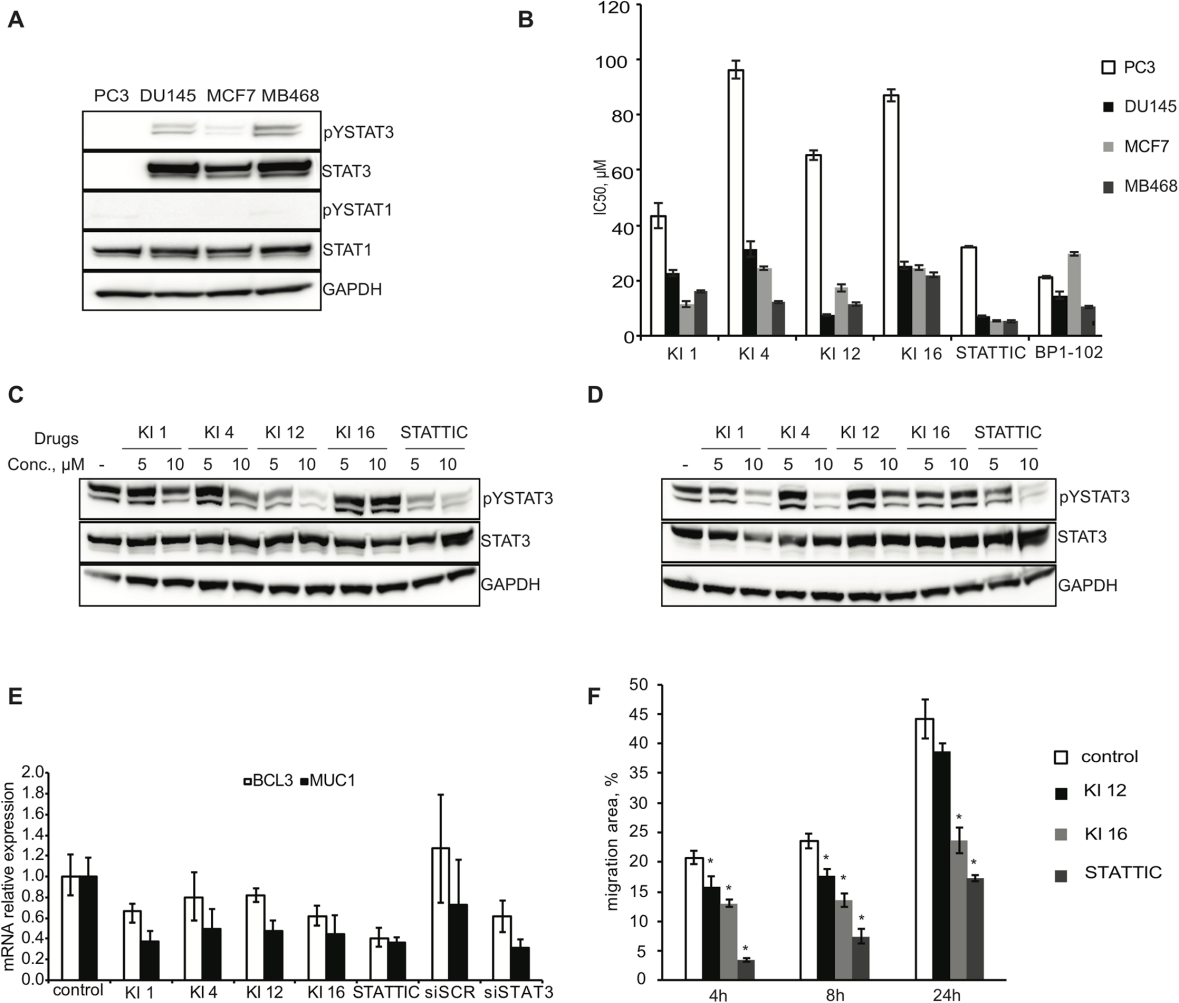
Differential effect of the lead compounds on viability of STAT3-dependent and STAT3-independent cancer cell lines. **(A)** Cell pellets from the prostate cancer cells lines PC3 and DU145, and the breast cancer cell lines MCF7 and MDA-MB468, were lysed and subjected to Western blotting analysis. Antibodies against pSerSTAT3, pTyrSTAT3, pTyrSTAT1 and the corresponding antibodies for the total levels of these proteins were used. GAPDH was used as a loading control. **(B)** PC3, DU145, MCF7 and MDA-MB468 cells were seeded in 384-well white plates using Multidrop. Cells were treated with the range of compounds concentration using Echo liquid dispenser. Cell viability was assessed after 48h using CellTiterGlo viability assay (Promega) according to the instructions of the manufacturer. The viability of the DMSO-treated cells was set as 100%. The data represents IC50 values for the four cell lines calculated from three replicates ± SD. **(C, D)** DU145 **(C)** and MDA-MB468 **(D)** cells were treated with the indicated compounds for 4 h. Levels of phospho-STAT3 and total STAT3 were determined by Western blotting analysis. GAPDH was used as a loading control. **(E)** DU145 cell line was transfected with 50 nM scrambled siRNA or STAT3 siRNA for 48 h or treated with the indicated compounds for 16 h and 24h, respectively. The concentrations of the compounds were taken from the IC_50_ values for viability (**3B**). Expression of the indicated STAT3 target genes was analysed by qRT-PCR. The values are normalized to the expression of GUSB and presented as a relative expression to untreated controls. Error bars represent mean + SE from three independent experiments. **(F**) A wound healing assay was performed using DU145 cells treated with the indicated compounds at 20 μM. Migration into the denuded area was monitored after 4, 8 and 24 h. The data represents mean of triplicates ± SE from three independent experiments. * p < 0.05.

To evaluate the specificity of the compounds for STAT-dependent cancer cells, we performed viability assays on two pairs of cell lines of prostate and breast cancer origin, with different basal STAT3 activity and differential dependence on STAT3 activity. The prostate adenocarcinoma cell line PC3 is STAT3 negative, whereas DU145 has constitutively activated pSTAT3, most probably due to an autocrine production of IL6 [51]. Breast adenocarcinoma MCF7 cells have low levels of pSTAT3 [52], while MDA-MB-468 cells express high basal levels of pSTAT3 and have also been shown to auto-secrete IL6 [53]. We have confirmed these data using Western blotting; phosphorylated STAT1 was not detected in either of these cancer cell lines and, therefore, was not further investigated (**Fig 5A**).

In a similar trend to a known STAT3 inhibitor BP1-102, the lead compounds preferentially impaired viability in at least one of the STAT3-dependent breast or prostate cancer cell lines (**Fig 5B)**. Compounds KI12 and KI16 showed a good selectivity profile further suggesting that these are promising inhibitors of STAT3 signaling and may have activity as potential cancer therapeutic agents.

We next tested the effect of the hit compounds towards the constitutively active pSTAT3 in DU145 and MDA-MB-468 cells. Compounds KI1 and KI4 could inhibit the constitutive levels of pSTAT3 in DU145 and MDA-MB-468 cells, especially at higher concentrations (**Fig 5C** and **5D**). This might be due to the previously mentioned similarity of these compounds to known kinase inhibitors and thus, inhibition of pSTAT3 downstream of TKs. KI12 was able to impair pSTAT3 in DU145 in line with the DU145 cell viability data. Surprisingly, KI16 did not affect constitutive pSTAT3 in these cell lines. This may indicate that longer treatments and/or higher drug concentrations are required to inhibit pSTAT3 in these cells.

The transcription of the STAT3-regulated *BCL3* and *MUC1* genes was investigated upon treatment with the compounds in the DU145 cells. Regulation of these genes by STAT3 was confirmed using RNAi against STAT3 (**Fig 5E**). All four compounds affected the transcription of these two genes confirming that the compounds affect STAT3 transcriptional activity. (**Fig 5E**).

Finally, we assessed the effects of compounds KI12 and KI16 on a STAT3-driven cancer-associated phenotype. Among many cancer-related processes, STAT3 has been shown to regulate cell migration and, hence, enhance the metastatic capability of prostate epithelium cancer cells. On the contrary, genetic or pharmacological inhibition of STAT3 made the cells less motile. Thus, the DU145 cell line was used to assess the effect of the compounds on cell migration using a wound healing assay [54]. As shown in **Fig 5F**, both KI12 and KI16 decreased the speed of the wound closure in DU145 cell line both after 8h and 24h of treatment in comparison to the mock-treated cells. Namely, KI16 led to significant, about 2-fold decrease in the inflicted wound closure comparing to control.

In conclusion, using a comprehensive approach, this screening system helped identify promising inhibitors of STAT3 signaling. In-depth analysis of hit compounds in chemical and biological assays demonstrated that the lead compound KI16 may be a promising agent to further develop towards a STAT3-targeted cancer therapeutic.

## Materials and methods

### Cell lines, culture conditions

Sublines of human adenocarcinoma cell line DLD1 A4 (with homologously deleted STAT3) and A4wt (A4 reconstituted with the wild-type variant of STAT3) were a kind gift of Zhenghe Wang, Genetics and Case Comprehensive Cancer Center, Case Western Reserve University. A4 and A4wt cell lines stably expressing a STAT3-inducible luciferase reporter were generated in the lab and were grown on 1 mg/mL of Hygromycin (Life Technologies AB Sweden). MCF7, MDA-MB468, PC3 and DU145 cell lines were all purchased from LGC Standards.

A4, A4wt, MCF7 and MDA-MB-468 cell lines were cultured in Dulbecco’s modified Eagle’s medium; PC3 and DU145 cells were cultured in RPMI 1640 medium; all supplemented with 10% heat-inactivated fetal bovine serum, 2 mM L-glutamine, 100 μg/mL streptomycin and 100 U/mL penicillin at 37°C in 5% CO_2_. All media and reagents were from HyClone, Thermo Fisher Scientific Inc., Sweden. The cells were routinely tested against mycoplasma using Plasmotest (# REP-PT2) from InVivoGen (USA).

### Drugs, treatments and reagents, and screening of the chemical library

If not indicated otherwise, the cells were seeded 24 hours before the treatment. For the screening, the cells were seeded in white 384-well plates (Perkin Elmer, Sweden) in the concentration 4000 cells/well using Multidrop Combi dispencer (Thermo Fisher Scientific). When cells were treated with IL6, they were always treated together with its soluble receptor (sIL6R) in the concentrations 50 and 100 ng/mL correspondingly (purchased from PeproTech Nordic). Interferon gamma (IFNγ) treatment was performed in the concentration 40 IU/mL (also purchased from PeproTech Nordic). STAT3 inhibitor STATTIC (STAT inhibitor 5) was used in the concentration 10 μM if not stated otherwise (purchased from Selleck Chemicals). JAK inhibitor Pyr6 was purchased from Merck Millipore (Germany) and was used in the concentration 1 μM. BP-1-102 was purchased from Merck Millipore (Germany) and used in the indicated concentrations. All chemicals were reconstituted and stored according to manufacturer’s recommendations.

The compound library, ENAMINE Drug-Like Diversity Set used for the screening, was a property of Karolinska High Throughput Center and contained 28,000 individual compounds dissolved in DMSO (produced by ENAMINE Ltd., Ukraine). The library used for the screening was an aliquot of the mother library, so that the compounds did not undergo multiple cycles of exposure to the air. Compound dispensing for the screening was performed using ECHO 555 acoustic liquid handler (Labcyte Inc., the USA). Primary screening was performed in a single replicate, using 10 μM concentration of the compounds dissolved in DMSO. Cells were seeded in 384-well plates using the Multidrop^TM^ (Thermo Fisher Scientific, Sweden). During the secondary screening, the compounds were tested for their ability to interfere with the assay readout, i.e. inhibit the activity of luciferase. For this, the compounds in duplicates at 10 μM concentration were added to IL6-pretreated cells, and the measurement of luciferase activity was performed directly after.

### Luciferase assay

The cells were plated as described in opaque 96- or 384-well plates suitable for cell culture (PerkinElmer Inc., The Netherlands). The day after, the luciferase reporter was induced with the indicated cytokine with or without addition of the inhibitors (for the treatment settings, see Fig. legends for each experiment). The luciferase activity was measured using SteadyLite^TM^ Plus reporter gene assay system (PerkinElmer Inc., The Netherlands). For the screening, EnVision plate reader (from PerkinElmer, the USA) was used to measure total luminescence per well. For the experiments performed outside KHTC, Centro LB 960 microplate luminometer was used (Berthold Technologies GmbH & Co).

### Plasmids and establishing stable lines

4X repeated SIE-element from *CFOS* promoter together with the complimentary oligonucleotide were synthetized by Eurofins MWG Operon. The oligo was designed with the cleaved restrictions sites for XhoI on the 5’-end and for HindIII on the 3’-end.

The sense sequence of the oligonucleotide: TCGAGGGTTCCCGTCAATGCATCAGGTTCCCGTCAATGCATCAGGTTCCCGTCAATGCATCAA; the complementary antisense sequence: AGCTTTGATGCATTGACGGGAACCTGATGCATTGACGGGAACCTGATGCATTGACGGGAACCTGATGCATTGACGGGGAACCC. The annealed oligo was subcloned into pGL 4.27 plasmid (Promega Biotech AB, Sweden) using the indicated restriction sites. The full insert sequence was verified by sequencing (Eurofins MWG Operon, Germany).

For the establishment of the stable cell lines, A4 and A4wt cell lines were seeded in in 6-well tissue culture plates. The next day, the cells were transfected with 2 μg of previously linearized pGL4.27-SIE plasmid or empty vector using GeneJuice transfection reagent (Merck Millipore, Germany) according to the manufacturer’s instructions. The selection of cells expressing the construct was performed using 1 mg/mL of Hygromycin B (Life Technologies, The Netherlands) and subsequently confirmed using IL6 stimulation and a luciferase assay. At least 10 clones were verified per construct and evaluated for the fold induction by IL6 comparing to control and for the best reproducibility and the highest Z’-factor.

### Quantitative real-time reverse transcriptase PCR (qRT-PCR)

RNA was extracted using a Qiagen RNeasy mini kit (Qiagen AB, Sweden) and treated with DNase (Ambion Turbo DNA-free, Life Technologies Corp., Sweden). Approximately 100 to 500 ng of DNase-treated RNA was used to generate cDNAs, using M-MLV reverse transcriptase (Life Technologies Corp., Sweden) and a mixture of oligoDt_18_ with nanomers (IDT technologies Inc., Belgium). qRT-PCR was carried out using KAPA 2G SyberGreen (Kapa Biosystems, USA) and the Applied Biosystems 7900HT platform with the following conditions: 95°C for 3 min, 95°C for 3 sec, and 60°C for 30 sec. The primers are indicated in Table 1.

**Table 1:** Primers used for qRT-PCR.

Gene	Forward primer	Reverse primer
MUC1	GCGTGAGTGATGTGCCATTT	ACAGCCAAGGCAATGAGATAGA
JUNB	TGGACGATCTGCACAAGATGAA	GTAGCTGCTGAGGTTGGTGTAA
TRIM15	AGCCAGCAAGTGAGCTTCTA	CATCTCTGGGAGGGTGAGTATTT
BCL3	ACATCGACGCAGTGGACATT	GGAGCTGCCGGAGTACATTT
IL15	AGTGCTTTCTCTTGGAGTTACA	CAAACTGTTGTTTGCTAGGATGA
CXCL10	CCATTCTGATTTGCTGCCTTATC	TACTAATGCTGATGCAGGTACAG
OAS2	AATCAGCGAGGCCAGTAATC	CAGCCATTGCCAGCATATTT
IFI35	ATGATGTCCAGCCAGTTGAG	TTCCTAGTCTTGCCAAAGAAGAT
IRF1	CATGAGACCCTGGCTAGAGATG	TCCGGAACAAACAGGCATCC
β-actin	AGGTCATCACCATTGGCAATGAG	CTTTGCGGATGTCCACGTCA
U48	AGTGATGATGACCCCAGGTA	TCCTTGGATGTGGTAGCCGTTTCT

The expression of the genes was normalized to the expression of β-actin (or to U48 where indicated); relative expression was calculated using ΔΔCt method according to the instructions of the User Bulletin #2 (Applied Biosystems).

### Protein extraction

To extract total proteins, cellular pellets were resuspended in a modified RIPA buffer (50 mM Tris-HCl pH 7.4, 150 nM NaCl, 1 mM EDTA, 1% NP-40 and 1% Glycerol) supplemented with a protease inhibitor cocktail and phosphoSTOP (Roche AB Scandinavia), 1 mM orthovanadate and 1 mM DTT, and kept on ice for 10 min. The samples were then centrifuged at 14,000 rpm at 4°C for 10 min. The supernatant solutions were transferred into new tubes and the protein concentrations were measured using the Bradford assay (Bio-Rad Laboratories AB, Sweden).

### Western blotting and antibodies

A total of 20–30μg of protein was loaded onto 4–12% Bis-Tris gels (NuPAGE, Life Technologies Corporation, Sweden). After transfer, the PVDF membranes were blocked in 5% Blotting Grade Blocker (Bio-Rad Laboratories AB, Sweden) in TBS supplemented with 0.1% Tween-20 (TBST, both from Merck Millipore, Germany) and incubated with the primary antibodies at 4°C overnight. After 1 h incubation with secondary antibodies (HRP-conjugated anti-mouse from Rockland Immunochemicals Inc., #20789 and HRP-conjugated anti-rabbit from Cell Signaling Technology Inc., USA, #7074), the proteins were detected by using an ECL solution (PerkinElmer Inc., Sweden). The following antibodies were from Cell Signaling Technology: STAT1(#9172, 1/1000), p-Y701-STAT1 (#58D6, 1/1000), STAT3 (#79D7, 1/1000), p-Y705-STAT3 (#D3A7, 1/1000), JAK1 (#3332, 1/1000), JAK2 (#3230, 1/1000). Anti-GAPDH antibody (ab9485, 1/5000) was purchased from Abcam; pJAK1 (#sc-16773-R, 1/500), pJAK2 (#sc-16566-R, 1/500)–from Santa Cruz Biotechnology.

#### Viability assays

2500–10,000 cells were plated in DMEM medium in transparent flat-bottomed 384- or 96-well plates. Twenty-four hours later, the drugs were added at different concentrations and left for the indicated time. 48h viability of the cells in response to the compounds was assessed using ApoToxGlo Assay (Promega Biotech AB, Sweden) or CellTiterGlo (Promega Biotech AB, Sweden) according to the manufacturer’s instruction.

#### Wound healing assay

The wound healing assay was performed on confluent DU145 cells in 6-well plates that were starved for 24h in growth medium containing 0.5% FBS. A wound was created in the cell monolayer using a sterile pipette tip. After washing, cells were treated with or without 20 μM of KI12, KI16 or STATTIC and allowed to migrate into the denuded area for 24 h. Wound closure was monitored at 10X magnification using a Olympus CKX41 microscope with a Lumenera Infinity-1 digital camera. Migration area was calculated by measuring the wound area using ImageJ. Each experiment was done in triplicates.

#### In silico docking

The top 67 compounds from our screening campaign were selected for analysis using GLIDE docking in the Maestro 10.0 software suite. Compounds were docked to the STAT3 crystal structure (PDB 1BG1). Waters with less than three hydrogen bonds to non-water molecules were removed, and the overall structure was strain-minimized prior to docking. Ligands were prepared for *in silico* analysis using the ligand preparation application and the docking grid was created using residues 702–709 of the opposing STAT3 monomer. Compounds were docked under extra precision with flexible ligand sampling and without constraints for hydrogen or metal bonding. Ligand interaction diagrams were generated using the Maestro 10.0 suite and images of the docked compounds were created using Pymol under educational license use.

#### Data analysis and curve fitting

Data analysis was performed using Microsoft Excel; curve fitting and IC50-values calculations were performed using GraphPad software.

## Discussion

Constitutive STAT3 activation is a major contributor to the pathogenesis of cancer. However, the development of STAT3 inhibitors is particularly challenging due to the absence of well-defined binding pockets and relatively large surfaces that mediate protein-protein interactions and STAT3 activity. In spite of this, a number of STAT3-inhibiting compounds have been presented in the scientific literature [15]. While great progress has been made within the field, a combination of low potency, poor specificity and inappropriate pharmacology has so far hindered the advancement of STAT3 inhibitors into clinical settings.

Because STAT3 can be activated by several different upstream kinases, developing an inhibitor that targets STAT3 directly could offer improved therapeutic effects and reduce the likelihood of acquired resistance. Thus, our screening campaign attempted to enrich for compounds that may directly bind to STAT3 protein. The timing scheme we used also reduced the likelihood of identifying JAK inhibitors as the potential inhibitors were added at a time point when JAKs are already deactivated and, thus, not sensitive to inhibition.

The selected hit compounds KI1, KI4, KI12 and KI16 all inhibited STAT3-dependent gene expression and preferentially reduced viability in cell lines that are dependent on high levels of STAT3 activity. Unexpectedly, compounds KI1 and KI4 showed close structural similarity to known EGFR and VEGFR inhibitors. Accordingly, these compounds inhibited IL6-mediated JAK1 and JAK2 phosphorylation pointing towards that the observed inhibition of STAT3 phosphorylation is likely due to the inhibition of upstream factors within the STAT3 signaling cascade. It is suspected that direct inhibition of EGFR could result in impaired signaling via the IL6 receptor, as these receptors are known to function cooperatively [55]. Thus, this may explain the observed inhibition of IL6-induced STAT3 activity by these compounds. Alternatively, as these molecules possess known adenine mimetics, they may also bind the ATP–pockets of other kinases. While these compounds are clearly potent inhibitors of STAT3 transcriptional activity, the complex and unclear mechanism of action, as well as similarity to known RTK inhibitors suggest that these would not be amenable to further optimization using medicinal chemistry efforts.

Compounds KI12 and KI16 had a more predictable profile as STAT3 pathway inhibitors and did not show strong inhibition of the upstream JAKs. Of these two compounds, KI16 had a good docking score when docked into the STAT3 SH2 domain. KI16 also inhibited IL6-induced STAT3 phosphorylation in A4wt cells, but not in cell lines with constitutively active STAT3, at least under studied conditions. KI12 could impair STAT3 phosphorylation in DU145 cells, however at concentrations well above those that were found to impair viability in these cells. KI12 and KI16 both impaired STAT3-dependent gene expression in the DU145 cell line. Furthermore, these compounds also showed selectivity for STAT3 inhibition over STAT1 as exemplified by preferential disruption of IL6-induced genes over IFNγ-induced genes in A4/A4wt cell lines. KI12 and KI16 also inhibited cell migration in the wound healing assay suggesting that the compounds can potentially interfere with STAT3-mediated pro-tumorigenic functions.

KI1, KI4, KI12 and KI16 also inhibited STAT1-dependent transcriptional activity. Although this was not an intended result, this was not a completely unexpected finding. STAT3 and STAT1 proteins are very similar in structure, size, mode of activation (phosphorylation and dimer formation through SH2-pTyr interactions) and the ability to bind to the same DNA consensus motifs. Therefore, it is reasonable that the inhibitors identified in this screening campaign could affect STAT1 and other STAT family members. This could explain some of the toxicity that is observed in the cell lines that do not depend on STAT3 phosphorylation (e.g., PC3, A4 and A4wt).

To conclude, we report the construction and optimization of a screening system for monitoring cytokine-induced STAT transcriptional activity. A high-throughput screen identified several hit compounds which were further tested in an array of assays to further characterize their effects on cells. KI16 that was selected as the top STAT3 pathway inhibitor represents a promising lead for further optimization and development into therapeutic agents and molecular tools to unravel novel mechanisms of STAT3-driven transcription.

## Supporting information

S1 Fig Characterization of the hit compounds. **(A)** A4wt cells were seeded in opaque 96-well plates, left overnight and then treated with the compounds in the indicated concentrations for 5h (as in the screening). The viability was assessed by Acid Phosphatase Assay. 100% is set to the DMSO-treated cells. **(B)** The Ct values for β-actin expression as measured by qRT-PCR in the samples from 4B. A4wt cells were treated with the compounds for 5 h, RNA was isolated and equal amount of RNA was used for cDNA synthesis.
(TIFF)

S2 Fig Lead compounds inhibit STAT1-dependent transcription of the genes. A4wt **(A)** and A4 cells **(B)** cells were pretreated with the indicated compounds for 30 min and then treated with IFNγ (40 IU/mL) for 4 h. RNA was extracted and the mRNA levels of the indicated STAT target genes were assessed by qRT-PCR. The expression is normalized to β-actin expression and is presented relative to untreated control. The data represents mean of duplicates + SD.
(TIFF)

S1 File Secondary screening results. The file includes information about the compounds that showed inhibition of STAT3 transcriptional activity in the secondary screening.
(XLSX)
