# Supplementary figures and images for: Identification of novel small molecules that inhibit STAT3-dependent transcription and function
Source: PLoS One. 2017 Jun 21;12(6):e0178844. doi: 10.1371/journal.pone.0178844 (PMC5479526; doi:10.1371/journal.pone.0178844)

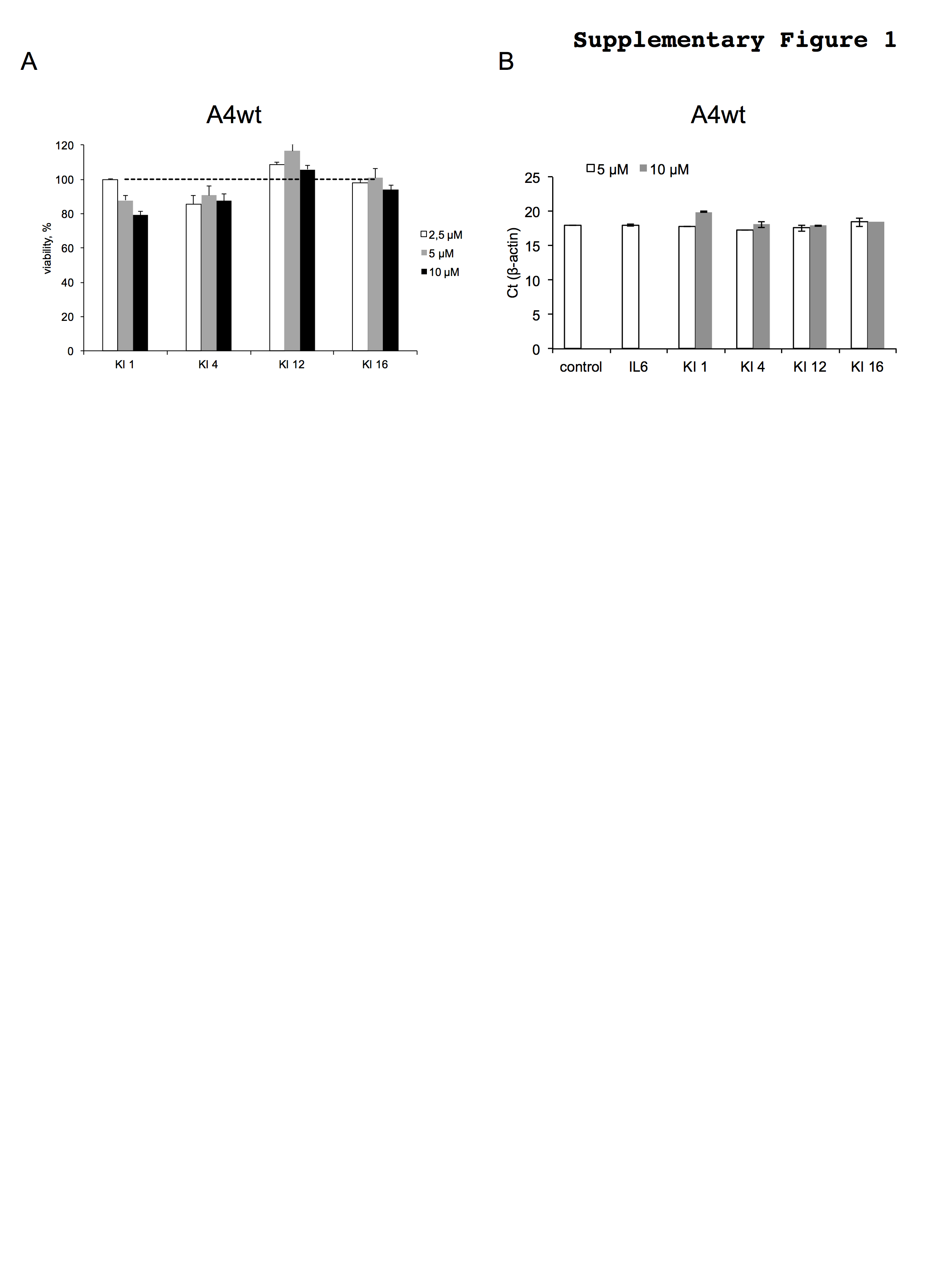

Supplement: S1 Fig — (A) A4wt cells were seeded in opaque 96-well plates, left overnight and then treated with the compounds in the indicated concentrations for 5h (as in the screening). The viability was assessed by Acid Phosphatase Assay. 100% is set to the DMSO-treated cells. (B) The Ct values for β-actin expression as measured by qRT-PCR in the samples from 4B. A4wt cells were treated with the compounds for 5 h, RNA was isolated and equal amount of RNA was used for cDNA synthesis. (TIFF) [file pone.0178844.s001.tiff]

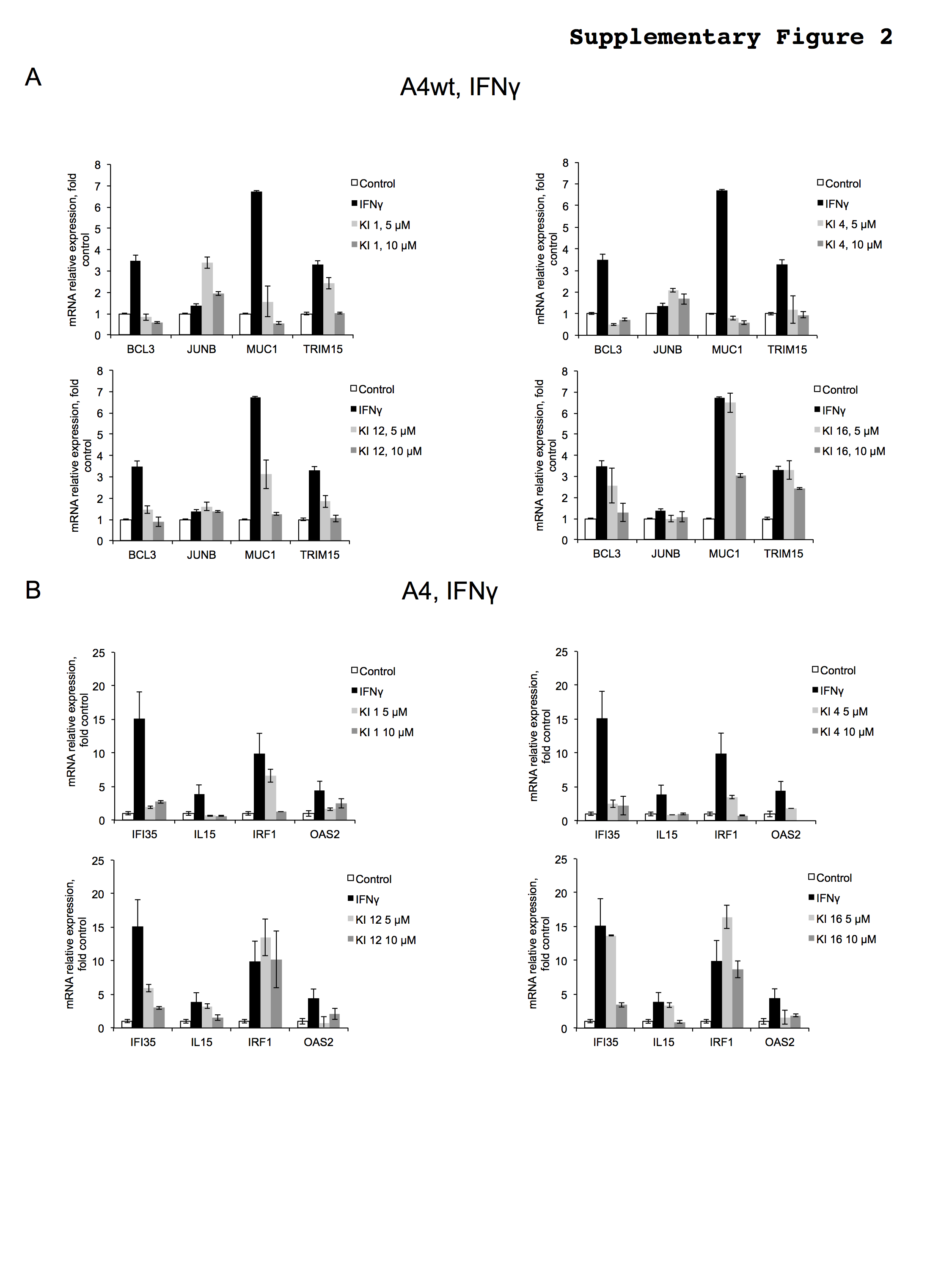

Supplement: S2 Fig — A4wt (A) and A4 cells (B) cells were pretreated with the indicated compounds for 30 min and then treated with IFNγ (40 IU/mL) for 4 h. RNA was extracted and the mRNA levels of the indicated STAT target genes were assessed by qRT-PCR. The expression is normalized to β-actin expression and is presented relative to untreated control. The data represents mean of duplicates + SD. (TIFF) [file pone.0178844.s002.tiff]
